# Quality of life, depression and anxiety in cerebral amyloid angiopathy: A cross‐sectional study

**DOI:** 10.1111/ene.16476

**Published:** 2024-09-22

**Authors:** Kanishk Kaushik, Natasha G. Waslam, Reinier G. J. van der Zwet, Sabine Voigt, Rosemarie van Dort, Erik W. van Zwet, Gisela M. Terwindt, Ellis S. van Etten, Marieke J. H. Wermer

**Affiliations:** ^1^ Department of Neurology Leiden University Medical Center (LUMC) Leiden The Netherlands; ^2^ Department of Radiology Leiden University Medical Center (LUMC) Leiden The Netherlands; ^3^ Department of Biomedical Data Sciences Leiden University Medical Center (LUMC) Leiden The Netherlands; ^4^ Department of Neurology University Medical Center Groningen (UMCG) Groninjen The Netherlands

**Keywords:** anxiety, cerebral amyloid angiopathy, depression, HRQoL

## Abstract

**Background and Purpose:**

Data on health‐related quality of life (HRQoL) and mood in cerebral amyloid angiopathy (CAA), a disease characterized by stroke and cognitive decline, are limited. We aimed to investigate the impacted domains of life, value‐based HRQoL and the prevalence of depression and anxiety in patients with CAA.

**Methods:**

We conducted a cross‐sectional study of patients with sporadic (s)CAA, lobar dominant mixed CAA and hypertensive arteriopathy (mixed CAA‐HTA), or Dutch‐type hereditary (D‐)CAA, from prospective outpatient clinic cohorts. Participants completed four questionnaires: the EuroQoL 5 dimensions 5‐level questionnaire (EQ‐5D‐5L; EQ‐VAS for visual analogue scale; EQ‐Index for index rating), the Short‐Form 36 questionnaire (SF‐36), the Center for Epidemiologic Studies—Depression scale (CES‐D), and the Hospital Anxiety and Depression Scale (HADS; ‐D for depression and ‐A for anxiety subscales). The EQ‐5D‐5L assesses the domains mobility, self‐care, usual activities, pain/discomfort and anxiety/depression. The SF‐36 domains are physical functioning, social functioning, physical role limitations, emotional role limitations, mental health, vitality, bodily pain, and general health perceptions. We compared age‐ and sex‐ adjusted HRQoL (SF‐36 domain scores; EQ‐VAS; EQ‐Index) to the Dutch normative population, and estimated the prevalences of current depression (either: history of depression or current use of antidepressants, with high score on CES‐D [≥16] and/or HADS‐D [≥8]; or high score on both depression questionnaires) and anxiety (HADS‐A ≥ 8).

**Results:**

We included 179 patients: 77 with sCAA (mean age: 72 years, women: 36%), 31 with mixed CAA‐HTA (68 years, women: 29%), and 71 with D‐CAA (56 years, women: 52%, symptomatic: 35 [49%]). The SF‐36 profiles of all patient groups were similar, negatively differing from the norm in emotional role functioning, social functioning and vitality. The EQ‐VAS score of patients (mean [SD] sCAA: 76 [16], D‐CAA: 77 [15]) was similar to the norm, as was the EQ‐Index score. Fifteen patients with sCAA (23%; 95% confidence interval [CI] 13%–33%), seven with mixed CAA‐HTA (27%; 95% CI 10%–44%) and eight with D‐CAA (14%; 95% CI 5%–22%) were noted as having depression. The prevalences of anxiety and depression were equivalent.

**Conclusions:**

We found that CAA influenced emotional role functioning and aspects linked to social engagement consistently across its subtypes. One quarter of patients exhibited depressive or anxiety symptoms. Recognizing these impacted domains could enhance overall well‐being.

## INTRODUCTION

Cerebral small‐vessel disease is associated with impaired health‐related quality of life (HRQoL) and a high prevalence of depression [1]. Cerebral amyloid angiopathy (CAA) is a common cerebral small‐vessel disease subtype caused by the accumulation of amyloid‐ß in the walls of cerebral vessels and is characterized by intracerebral hemorrhage (ICH), cognitive decline, and transient focal neurological episodes (TFNE) [2].

There are different subtypes of CAA. The common sporadic form of CAA (sCAA) is per definition only present in patients aged ≥50 years [3]. Dutch‐type hereditary CAA (D‐CAA), a genetic variant caused by a point mutation of the *APP* gene, has an onset two decades earlier than sCAA and is characterized by a similar disease course, but with less age‐related co‐pathology [2]. Patients with radiological evidence of CAA with concomitant hypertensive arteriopathy (mixed CAA‐HTA) share clinical similarities to patients with sCAA but might have a different prognosis [4].

Although the disease manifestations of CAA may be severe, no studies have been conducted on sporadic or hereditary CAA to assess their impact on the domains of life or on value‐based HRQoL. Value‐based HRQoL pertains to the valuation of the combination of a person's overall physical, mental, and social well‐being, related to an individual's health or affected by the presence of disease, and not merely the absence of disease and infirmity [5, 6].

Similarly, knowledge of the impact of CAA on mood is limited [7]. Informant‐reported depression might be present in 15% up to 49% of patients and anxiety in 7%–38% [8, 9, 10]. In addition, survivors of CAA‐related ICH have been suggested to be more likely to experience depressive symptoms than hypertension‐related ICH survivors [11, 12]. Importantly, knowledge about patient‐reported depressive symptoms and anxiety is limited and not generalizable to the entire CAA population, which also consists of patients without ICH. Furthermore, depressive and anxiety symptoms have not been systematically described in (pre)symptomatic D‐CAA, and theoretically may be different compared to sCAA due to the hereditary nature of D‐CAA with patients that have experiences with other cases in the family [13].

In this study we investigated: (i) the impacted domains of life; (ii) value‐based HRQoL; and (iii) prevalence of depression and anxiety in patients with sCAA, mixed CAA‐HTA and (pre)symptomatic D‐CAA.

## METHODS

### Patient selection

We included patients from our prospective CAA cohorts comprising patients who presented (January 2012–September 2022) at the CAA (out)patient clinic of the Leiden University Medical Center (LUMC; a CAA referral center in the Netherlands), or at the LUMC (out)patient clinic for monogenetic forms of cerebral hereditary angiopathies. These prospective CAA cohorts included patients diagnosed with sCAA, mixed CAA‐HTA (with predominantly lobar hemorrhage distribution pattern), or D‐CAA. This cross‐sectional observational study was conducted between July 2021 and November 2022.

Patients with sCAA were all diagnosed as ‘probable CAA’ according to the modified Boston criteria. Because vascular risk factors are often present in the elderly, we included patients who met the modified Boston criteria except for the presence of concomitant deep cerebral microbleeds but with a predominantly lobar hemorrhage distribution pattern (mixed CAA‐HTA) and analyzed them separately. Predominantly lobar hemorrhage distribution was defined as either 1: ≥10 deep: lobar cerebral microbleeds; or cortical superficial siderosis and 1: <10 deep: lobar cerebral microbleeds [14]. We did not assess other non‐CAA related small‐vessel disease markers, such as basal ganglia enlarged perivascular spaces, when classifying patients as mixed CAA‐HTA. Participants with (pre)symptomatic D‐CAA were aged ≥18 years and had either (i) a DNA‐proven mutation of codon 693 of the *APP* gene, or (ii) a medical history of ≥1 symptomatic ICH (sICH) with ≥1 first‐degree relative with DNA‐confirmed D‐CAA. Patients with D‐CAA with previous sICH were considered symptomatic.

Patients were ineligible if (i) no current postal address or e‐mail address was known, or if (ii) they were not willing or able to fill out the study questionnaires. Eligible patients were informed through written information and a telephone call and asked to fill out a cross‐sectional questionnaire package sent via e‐mail or post as preferred. Patients were included if informed consent was obtained. Patients who did not fill out the questionnaire within 10 days were reminded once via telephone or e‐mail. Patients who then did not return any questionnaires were excluded from the analyses (non‐responders).

This study was approved by the LUMC Medical Ethics Committee, which concluded that it did not fall under the Medical Research on Human Subjects Act (non‐WMO; protocol: N21.062). In accordance with the Declaration of Helsinki, all patients provided written or electronic informed consent. This study followed the Strengthening the Reporting of Observational Studies in Epidemiology (STROBE) guidelines.

### Data collection

#### Clinical data

Data on demographics, medical history, and clinical symptoms including history of sICH and self‐reported depression, were collected from the most recent outpatient clinic visit in the electronic patient files prior to the date of questionnaire administration. Information about level of education, marital status, and body mass index (BMI; kg/m^2^) was collected in the questionnaire package, which also contained the four study questionnaires (all collected at the same time).

#### Health‐related quality of life

We used the Short‐Form 36 (SF‐36) and the EuroQOL 5 dimensions, 5‐level questionnaire (EQ‐5D‐5L) to measure HRQoL [15, 16].

The SF‐36 consists of 36 questions assessing eight domains of life: physical functioning; social functioning; role limitations due to physical problems (physical role limitations); role limitations due to emotional problems (emotional role limitations); mental health; vitality; bodily pain; and general health perceptions. It also asks for perceived changes in health status in the past 12 months. Scoring was carried out according to the manual: scores on each domain are coded, summed and transformed to a 0–100 scale (worst possible to best possible health state) [15, 17]. For comparison with the Dutch population, we used the national sample norms from previous literature [18].

The EQ‐5D‐5L assesses five domains (mobility, self‐care, usual activities, pain/discomfort, anxiety/depression) through one question with five ordered response categories (no problems, slight problems, moderate problems, severe problems, unable). Scoring was conducted according to the user manual [16, 19]. Using this questionnaire different health states can be described. Participants also rate self‐perceived health on that day with a visual analogue scale (EQ‐VAS), which ranges from 0 to 100 (worst to best imaginable health). The value‐based EQ‐Index score is calculated by transforming the health states with a population‐weighted utility value as previously published [19, 20]. For the Dutch population, this score ranges from −0.446 to 1, where 0 corresponds to ‘being dead’, 1 to ‘perfect health’, and negative values are considered ‘worse than dead’. Permissions for the academic use of the EQ‐5D‐5L were obtained from the EuroQOL Group (Registration: 39323).

#### Depressive symptoms and anxiety

We administered the Centre for Epidemiological Studies Depression scale (CES‐D), a 20‐item validated self‐reported 4‐point scale (0 = rarely or never [<1 day/week]; 1 = some or a little of the time [1–2 days/week]; 2 = occasionally or a moderate amount of time [3–4 days/week]; 3 = most or all of the time [5–7 days/week]), to assess depressive symptoms in the past week [21]. The responses are summed, with a higher score indicative of greater depressive symptoms. A score of ≥16 was indicative of depressive symptoms [22].

In addition, we used the Hospital Anxiety and Depression Scale (HADS): a 14‐item validated self‐reported 4‐point scale to screen for presence of depression or anxiety. It consists of two scales, HADS‐A for anxiety and HADS‐D for depression, with seven items [23]. We used a HADS‐D or HADS‐A score of ≥8 as indicative of depressive symptoms or anxiety [24].

We marked patients to have current depressive symptoms (which we further refer to as being depressed) if they had either (i) a medical history of depression or current use of antidepressant medication, with a high score on the CES‐D (≥16) and/or HADS‐D (≥8); or (ii) a high score on both CES‐D and HADS‐D. We estimated the prevalence of current depression accordingly.

#### Statistical analysis

Data are presented for the patients with sCAA, mixed CAA‐HTA, and D‐CAA (all, stratified by pre‐ and symptomatic) separately. Demographic data, clinical characteristics, and frequencies are displayed as mean with standard deviation (SD), median with interquartile range (IQR), or *n* (%) as appropriate.

We present descriptive statistics of the SF‐36 domain scores. We used generalized linear regression to compare the SF‐36 domain scores of patients (sCAA/D‐CAA) with the Dutch norms, entering the patient age‐ and sex‐specific norm score as offset. We present descriptive EQ‐5D‐5L health states. We calculated the age‐ and sex‐adjusted difference between the EQ‐Index scores of patients (sCAA/mixed CAA‐HTA/D‐CAA) and the normative population. A priori, we defined an EQ‐Index score difference of ≥0.100 to be clinically relevant [25]. We used generalized linear regression to compare the EQ‐VAS scores of patients (sCAA/mixed CAA‐HTA/D‐CAA) with the Dutch norms, entering the patient age‐ and sex‐specific norm score as offset. We present the proportion of patients marked as depressed as well as the median and dichotomous CES‐D and HADS data. We present the proportion marked to have anxiety based on the HADS‐A.

Missing data were addressed through mean imputation according to prespecified assumptions concerning mechanism of missingness (Supplemental Methods) [26, 27]. We performed a complete‐case sensitivity analysis to assess the impact of the imputation on the HADS and CES‐D scores. We assessed generalizability by comparing the demographic and clinical characteristics of included and excluded patients in another sensitivity analysis.

## RESULTS

In total, 179 (86%) of the 208 questionnaire packages were returned by 77 patients with sCAA (mean age 72 years, 36% women), 31 with mixed CAA‐HTA (age 68 years, 29% women), and 71 with D‐CAA (age 56 years, 52% women; Table 1; Figure S1). In patients with sCAA, 36 patients (47%) had previously presented with sICH, 31 (40%) with cognitive decline, 22 (29%) with TFNE, and 4 (5%) with epilepsy. These proportions were similar in patients with mixed CAA‐HTA (39% sICH, 48% cognitive decline, 6% TFNE, 10% epilepsy) and D‐CAA (49% sICH, 35% cognitive decline, 7% TFNE, 11% epilepsy).

**TABLE 1:** Baseline characteristics.

	sCAA	Mixed CAA‐HTA	D‐CAA		
	All (*n* = 77)	All (*n* = 31)	All (*n* = 71)	Symptomatic (*n* = 35)	Presymptomatic (*n* = 36)
Age, years, mean (SD)	72 (6)	68 (7)	56 (13)	63 (9)	49 (13)
Men, *n* (%)	49 (64)	22 (71)	34 (48)	17 (49)	17 (47)
Education, years, mean (SD)^a^	14 (4)	16 (4)	13 (3)	13 (4)	13 (3)
Education, level, high, *n* (%)	25 (33)	12 (39)	21 (30)	12 (34)	9 (25)
Medical history, *n* (%)					
sICH	36 (47)	12 (39)	35 (49)	35 (100)	–
Cognitive decline	31 (40)	15 (48)	25 (35)	18 (51)	7 (19)
TFNE	22 (29)	2 (6)	5 (7)	5 (14)	0 (0)
Epilepsy	4 (5)	3 (10)	8 (11)	7 (20)	1 (3)
Hypertension	37 (48)	16 (52)	23 (32)	12 (34)	11 (31)
Previous sICH count, median (IQR)	0 (0–1)	0 (0–1)	0 (0–2)	2 (1–3)	–
Use of antidepressant medication, *n* (%)	5 (6)	1 (3)	4 (6)	2 (6)	2 (6)
Elapsed time since event, median (IQR)					
Diagnosis, years	2 (1–3)	2 (0–3)	5 (3–8)	5 (2–9)	4 (3–8)
Last outpatient clinic visit, months	6 (2–12)	9 (4–25)	7 (2–25)	5 (2–11)	8 (1–33)
Previous sICH^b^, years	3 (1–4)	4 (4–5)	3 (2–6)	3 (2–6)	–
Recruitment method, *n* (%)					
E‐mail	56 (73)	25 (81)	60 (85)	28 (80)	37 (90)
Post	21 (27)	6 (19)	11 (16)	7 (20)	4 (10)
Marital status^c^					
Unmarried	12 (16)	1 (3)	16 (23)	7 (20)	9 (25)
Married	45 (58)	16 (52)	37 (52)	20 (57)	17 (47)
Divorced	3 (4)	1 (3)	1 (1)	0 (0)	1 (3)
Widowed	4 (5)	0 (0)	3 (4)	2 (6)	1 (3)
Cigarette smoking, *n* (%)^d^					
Never	24 (31)	17 (55)	31 (44)	16 (46)	15 (42)
Past	46 (60)	10 (32)	29 (41)	15 (43)	14 (39)
Current	5 (6)	3 (10)	7 (10)	1 (3)	6 (17)
Alcohol, units/week^e^					
0	36 (47)	13 (42)	43 (61)	20 (57)	23 (64)
≤14	33 (43)	14 (45)	20 (28)	9 (26)	11 (31)
>14	6 (8)	2 (6)	2 (3)	1 (3)	1 (3)
BMI, kg/m^2^, mean (SD)^f^	25 (3)	27 (4)	28 (5)	27 (4)	30 (7)

Abbreviations: BMI, body mass index; D‐CAA, Dutch‐type cerebral amyloid angiopathy; IQR, interquartile range; sCAA, sporadic cerebral amyloid angiopathy; mixed CAA‐HTA, cerebral amyloid angiopathy with concomitant hypertensive arteriopathy; (s)ICH, (symptomatic) intracerebral hemorrhage; TFNE, transient focal neurological episode.

^a^ Years of education years missing 72%, level missing 4%.

^b^ Recorded only for patients with history of sICH.

^c^ Marital status 22% missing.

^d^ Cigarette smoking 4% missing.

^e^ Alcohol 6% missing.

^f^ BMI 67% missing. Other variables no missing.

### Health‐related quality of life

#### Short‐Form 36 questionnaire

**FIGURE 1:**
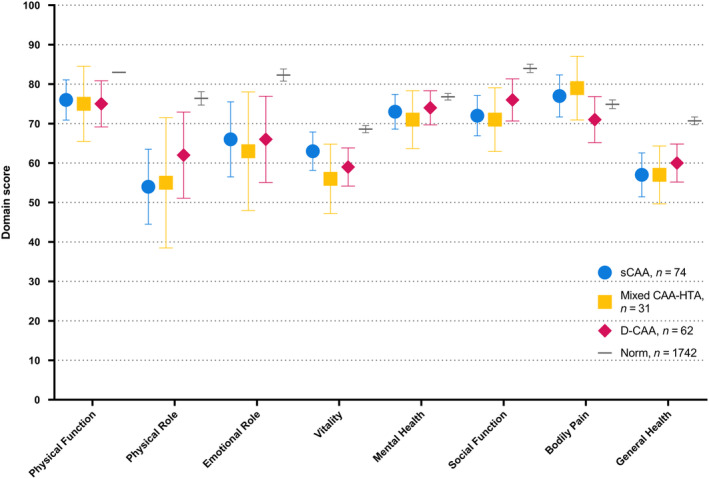
Short‐Form 36 domain scores of patients with sporadic cerebral amyloid angiopathy (sCAA), lobar dominant mixed cerebral amyloid angiopathy and hypertensive arteriopathy (CAA‐HTA), and hereditary Dutch‐type cerebral amyloid angiopathy (D‐CAA), compared to a Dutch normative sample Presented as mean (95% confidence interval). Normative population derived from previously published data [18].

The SF‐36 profiles of patients with sCAA, mixed CAA‐HTA, and D‐CAA were similar (Table 2, Figure 1). Patients with sCAA scored worse than the normative population in the domains of role functioning due to emotional problems (adjusted [adj.] β: −11 [95% CI −22;−1]) and social functioning (adj. β: −7 [95% CI −12;−1]), and better on the domains of physical functioning (adj. β: 11 [95% CI 5;17]) and bodily pain (adj. β: 8 [95% CI 2;14]). Similarly, patients with mixed CAA‐HTA scored worse than the norm in the domains of role functioning due to emotional problems, vitality, and social functioning (Supplemental Results). Patients with D‐CAA scored worse than the normative population in the domains of role functioning due to emotional problems (adj.β: −14 [95% CI −25;−2]) and vitality (adj.β: −7 [95% CI −13;−1]), and similar to the norm in all other domains.

**TABLE 2:** SF‐36 domain scores of patients with sCAA, D‐CAA and mixed CAA‐HTA.

	sCAA (*n* = 77)	Mixed CAA‐HTA (*n* = 31)	D‐CAA (*n* = 71)	Normative population^a^ (*n* = 1742)
Responded, *n* (%)	74 (96)	31 (100)	62 (87)	–
SF‐36 subscales, mean (SD)^b^				
Physical functioning	76 (22)	75 (26)	75 (23)	83 (23)
Role limitations due to physical problems	54 (41)	55 (45)	62 (43)	76 (36)
Role limitations due to emotional problems	66 (41)	63 (41)	66 (43)	82 (33)
Vitality	63 (21)	56 (24)	59 (19)	69 (19)
Mental health	73 (19)	71 (20)	74 (17)	77 (17)
Social functioning	72 (22)	71 (22)	76 (21)	84 (22)
Bodily pain	77 (23)	79 (22)	71 (23)	75 (23)
General health	57 (24)	57 (20)	60 (19)	71 (21)
Health change	52 (26)	51 (19)	46 (25)	NA
SF‐36 Summary scales, mean (95% CI)^c^				
Physical components scale	42 (40–44)	42 (38–45)	41 (38–43)	50 (48–52)
Mental components scale	47 (44–49)	45 (42–49)	47 (45–50)	49 (47–52)

*Note*: See Tables S1 and S2 and Figures S2–S4 for analysis stratified by cerebral amyloid angiopathy‐related medical history.

Abbreviations: CI, confidence interval; D‐CAA, Dutch‐type cerebral amyloid angiopathy; mixed CAA‐HTA, cerebral amyloid angiopathy with concomitant hypertensive arteriopathy; sCAA, sporadic cerebral amyloid angiopathy; SF‐36, Short‐Form 36 questionnaire.

^a^ Previously published data by other authors [18].

^b^ Imputed data: role physical/social functioning/bodily pain 3%, role emotional/mental health/general health 4%, vitality 5%. Physical function and health change no missing data. Component scales 6% imputed.

^c^ Norms derived from the normative population [18], coefficients derived from scoring manual [17].

The domain scores of patients with symptomatic and presymptomatic D‐CAA were similar across all domains, although symptomatic patients tended to score numerically lower than their presymptomatic counterparts (Figure S2). The SF‐36 scores of patients stratified by medical history of sICH, cognitive decline, or TFNE were all similar (Tables S1 and S2, Figures S2–S4).

#### EuroQOL 5 dimensions, 5‐level questionnaire

**FIGURE 2:**
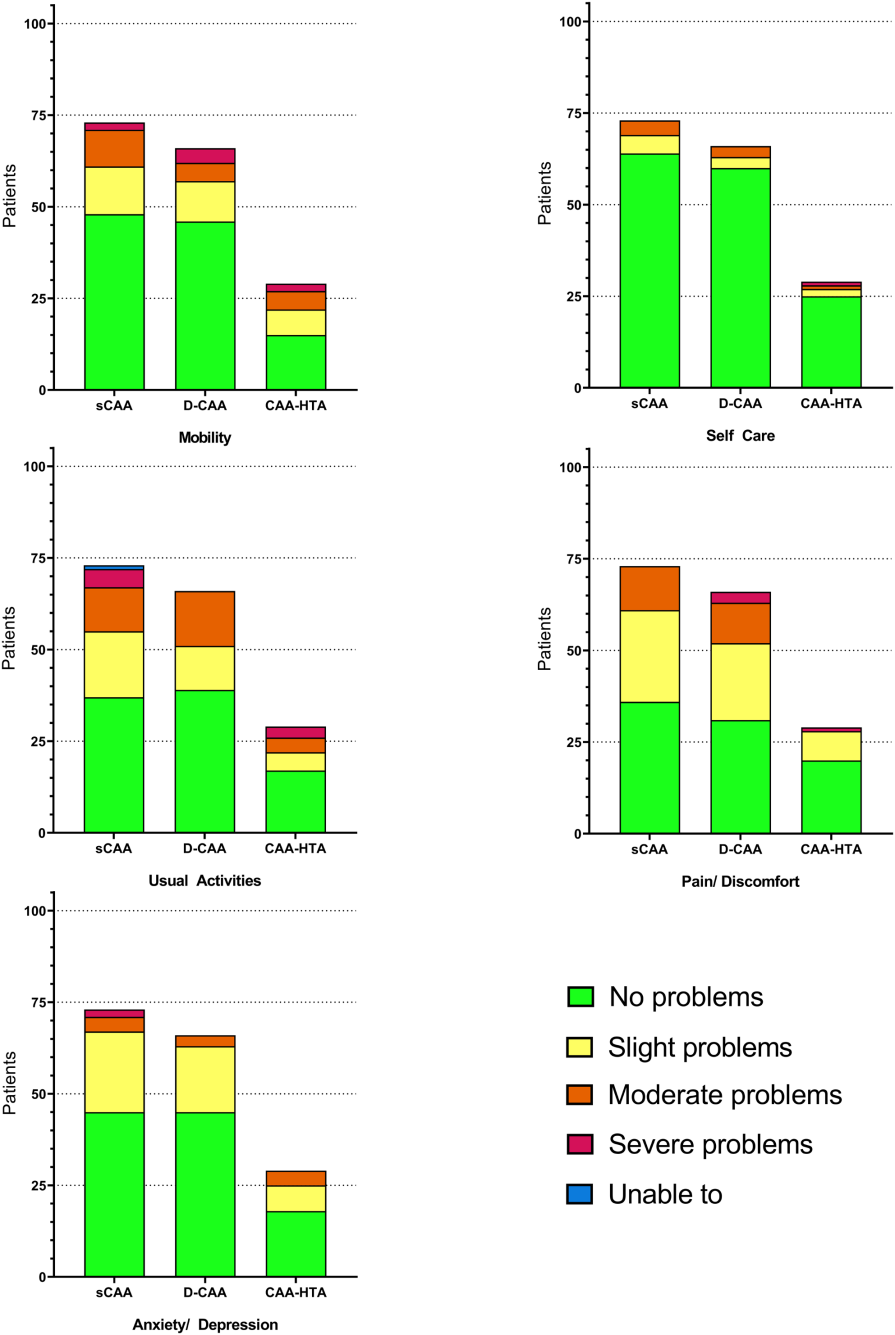
The EuroQoL 5 dimensions 5‐level health profile of patients with sporadic cerebral amyloid angiopathy (sCAA), Dutch‐type cerebral amyloid angiopathy (D‐CAA), and lobar dominant mixed cerebral amyloid angiopathy and hypertensive arteriopathy (CAA‐HTA).

The EQ‐5D‐5L health profiles of patients with sCAA, mixed CAA‐HTA, and D‐CAA were similar (Figure 2, Table 3). The most affected domains (moderate problems or worse) in sCAA were usual activities (*n* = 18, 25%) and mobility (*n* = 12, 16%). In D‐CAA, the most affected domains were usual activities (*n* = 15, 23%) and pain/discomfort (*n* = 14, 22%). In all groups, the least affected domain was self‐care (5% sCAA, 3% mixed CAA‐HTA, 5% D‐CAA). The health profiles and summary statistics were similar when stratifying patients by history of sICH, cognitive decline, or TFNE (Table S3 and Figures S5–S7).

**TABLE 3:** Health profile of patients with sCAA, D‐CAA and mixed CAA‐HTA.

	sCAA	Mixed CAA‐HTA	D‐CAA		
	All (*n* = 77)	All (*n* = 31)	All (*n* = 71)	Symptomatic (*n* = 35)	Presymptomatic (*n* = 36)
Responded, *n* (%)	73 (95)	29 (94)	66 (92)	33 (94)	33 (92)
EQ‐VAS score, mean (SD)	76 (16)	76 (20)	77 (15)	77 (17)	77 (12)
EQ index score, mean (SD)	0.82 (0.15)	0.82 (0.22)	0.84 (0.16)	0.81 (0.18)	0.86 (0.14)
Mobility, *n* (%)					
No problems	48 (66)	15 (52)	46 (70)	22 (67)	24 (73)
Slight problems	13 (18)	7 (24)	11 (17)	5 (15)	6 (18)
Moderate problems	10 (14)	5 (17)	5 (8)	2 (6)	3 (9)
Severe problems	2 (3)	2 (7)	4 (6)	4 (12)	–
Unable to walk about	0 (0)	0 (0)	0 (0)	–	–
Self‐care, *n* (%)					
No problems	64 (88)	25 (86)	60 (91)	29 (88)	31 (94)
Slight problems	5 (7)	2 (7)	3 (5)	2 (6)	1 (3)
Moderate problems	4 (5)	1 (3)	3 (5)	2 (6)	1 (3)
Severe problems	0 (0)	1 (3)	0 (0)	–	–
Unable to wash or dress	0 (0)	0 (0)	0 (0)	–	–
Usual activities, *n* (%)					
No problems	37 (51)	17 (59)	39 (59)	26 (79)	13 (39)
Slight problems	18 (25)	5 (17)	12 (18)	3 (9)	9 (27)
Moderate problems	12 (16)	4 (14)	15 (23)	4 (12)	11 (33)
Severe problems	5 (7)	3 (10)	0 (0)	–	–
Unable to do usual activities	1 (1)	0 (0)	0 (0)	–	–
Pain/discomfort, *n* (%)					
None	36 (49)	20 (69)	31 (47)	17 (51)	14 (42)
Slight	25 (34)	8 (28)	21 (32)	7 (21)	14 (42)
Moderate	12 (16)	0 (0)	11 (17)	7 (21)	4 (12)
Severe	0 (0)	1 (3)	3 (5)	2 (6)	1 (3)
Extreme	0 (0)	0 (0)	0 (0)	–	–
Anxiety/depression, *n* (%)					
None	45 (62)	18 (62)	45 (68)	23 (70)	22 (67)
Slight	22 (30)	7 (24)	18 (27)	8 (24)	10 (30)
Moderate	4 (5)	4 (14)	3 (5)	2 (6)	1 (3)
Severe	2 (3)	0 (0)	0 (0)	–	–
Extreme	0 (0)	0 (0)	0 (0)	–	–

*Note*: See Table S3 and Figures S5–S7 for analysis stratified by medical CAA‐related history (intracerebral hemorrhage /cognitive decline/ transient focal neurological episodes). All EQ‐5D variables ≤3% missing data.

Abbreviations: D‐CAA, Dutch‐type cerebral amyloid angiopathy; mixed CAA‐HTA, cerebral amyloid angiopathy with concomitant hypertensive arteriopathy; sCAA, sporadic cerebral amyloid angiopathy; VAS, visual analogue scale.

The mean EQ‐Index score of patients with sCAA was 0.82 (SD 0.15), which was similar to those with mixed CAA‐HTA (0.82 [SD 0.22]). The mean EQ‐Index score of patients with D‐CAA was 0.84 (SD 0.16), being similar in symptomatic and presymptomatic patients. The EQ index scores of patients were not different from the normative population (difference of means: sCAA −0.03, mixed CAA‐HTA −0.04, D‐CAA −0.04) and did not exceed the predefined threshold for clinical relevance (≥0.100). The age‐ and sex‐adjusted EQ‐VAS scores of patients with CAA (mean [SD] score sCAA: 76 [17]; D‐CAA 79 [14]) were not different from the normative population (adj. β value sCAA: −1 [95% CI −5;3]; mixed CAA‐HTA: −2 [95% CI −8;4]; D‐CAA: −3 [95% CI −7;1]).

#### Depression and anxiety

The combined prevalence of current depression was estimated at 23% (95% CI 13%;33%; *n* = 15) in sCAA, 27% (95% CI 10%;44%; *n* = 7) in mixed CAA‐HTA, and 14% (95% CI 5%;22%; *n* = 8) in D‐CAA (Table 4). These prevalences did not differ amongst patients with CAA when stratified by medical history of sICH, cognitive decline or TFNE (Table S3).

**TABLE 4:** Prevalence of depression in patients with sCAA, D‐CAA and mixed CAA‐HTA.

	sCAA	Mixed CAA‐HTA	D‐CAA		
	All (*n* = 77)	All (*n* = 31)	All (*n* = 71)	Symptomatic (*n* = 35)	Presymptomatic (*n* = 36)
Patients with sufficient data, *n* (%)	66 (86)	26 (84)	59 (83)	29 (83)	30 (83)
Depressed, *n* (%, 95% CI)	15 (23, 13–33)	7 (27, 10–44)	8 (14, 5–22)	3 (10, 0–21)	5 (17, 3–30)
History of depression or use of psychoactive medication, and high on CESD/HADS‐D	6 (9)	3 (12)	4 (7)	0 (0)	4 (13)
High score on both CESD and HADS‐D	13 (20)	6 (23)	6 (9)	3 (10)	3 (10)
CES‐D^a^					
Total score, median (IQR)	11 (4–18)	15 (8–21)	8 (5–18)	10 (5–15)	7 (4–18)
Scores high, *n* (%)	24 (36)	12 (46)	16 (27)	7 (24)	9 (30)
HADS‐D^a^					
Total score, median (IQR)	5 (2–8)	5 (2–8)	4 (1–6)	4 (3–6)	3 (1–5)
Scores high, *n* (%)	19 (28)	7 (26)	7 (12)	4 (13)	3 (11)
HADS‐A^a^					
Total score, median (IQR)	4 (1–7)	5 (3–8)	5 (2–7)	5 (2–7)	5 (2–8)
Scores high, *n* (%)	16 (23)	7 (26)	14 (23)	6 (19)	8 (29)

Abbreviations: D CES‐D, Center for Epidemiological Studies Depression Scale; D‐CAA, Dutch‐type cerebral amyloid angiopathy; HADS, Hospital Anxiety and Depression Scale (A for Anxiety subscale; D for depression subscale); IQR, interquartile range; mixed CAA‐HTA, cerebral amyloid angiopathy with concomitant hypertensive arteriopathy; sCAA sporadic cerebral amyloid angiopathy.

^a^ Scores after mean imputation, similar scores observed in complete‐case analysis (Table S5); Responded CES‐D/HADS: *n* = 66/69 sCAA, *n* = 64/65 D‐CAA, *n* = 26/27 mixed CAA‐HTA.

Anxiety symptoms (HADS‐A) were present in 16 (23%; 95% CI 13%;33%) patients with sCAA, seven (26%; 95% CI 9%;43%) with mixed CAA‐HTA, and 14 (23%; 95% CI 13%–34%) with D‐CAA (Table 4). Nine patients with sCAA, two with mixed CAA‐HTA, and five with D‐CAA were noted to have both depression and anxiety.

#### Sensitivity analyses

Included and excluded patients (*n* = 92: 63 excluded, 29 non‐responders) had similar baseline characteristics (Table 1, Table S4). There were no meaningful differences between the results of the complete‐case and mean‐imputed analyses of the CES‐D (8.6% imputed) and HADS groups (3.5% imputed; Table S5).

## DISCUSSION

We found that the health profiles of patients with sCAA, mixed CAA‐HTA and D‐CAA were comparable, most notably with limitations related to emotional functioning, social functioning, vitality and usual activities. These profiles were comparable across all domains, regardless of CAA‐related medical history, with a difference only for patients with a history of ICH who had worse physical role functioning. The value‐based HRQoL was not different from the normative population. Finally, up to one quarter of patients had depressive symptoms or anxiety.

Our findings on both HRQoL questionnaires complement each other. The SF‐36 demonstrated that patients had difficulties in role functioning due to emotional problems, social functioning, and vitality. This was in accordance with the most impacted EQ‐5D domain, which measures activities and social participation such as housework, leisure activities and work/study [28]. These quality‐of‐life impairments may hamper social participation, independent living and workforce participation of patients. Particularly noteworthy are patients with pre‐symptomatic D‐CAA, representing an early stage of the disease typically affecting those in the workforce or early stages of parenthood, who reported challenges in these areas. In addition, impairments in these domains might negatively impact cognitive reserve [29, 30, 31]. Furthermore, engaging in work or leisure activities is an important factor for improving quality of life [32]. Therefore, recognizing and addressing (modifiable) impairments related to usual activities and social participation in patients with CAA, especially the early stages of CAA, might enhance long‐term subjective well‐being, life satisfaction and self‐perceived HRQoL [31, 32, 33, 34]. In the longer term, developing a CAA‐specific HRQoL questionnaire, for example, through context‐independent international collaboration, might aid in the monitoring of such enhancements.

Surprisingly, patients with sCAA scored better than the normative population in the domain of physical functioning (adj.β: 11 [95% CI 5–17]) and bodily pain (adj.β: 8 [95% CI 2–14]), which was not observed in mixed CAA‐HTA or D‐CAA. However, baseline characteristics (except for age in D‐CAA) were similar in all three groups. Moreover, on the EQ‐5D‐5L, the proportion of patients reporting moderate or worse pain/discomfort was comparable between sCAA (*n* = 12; 16%) and D‐CAA (*n* = 14; 22%), both higher than in mixed CAA‐HTA (*n* = 1; 3%). This discrepancy might arise because the SF‐36 weighs both intensity of bodily pain and its interference in normal work, whereas the EQ‐5D‐5L only assesses pain intensity. Therefore, it remains unclear whether this finding reflects a true difference of patients with sCAA from the norm, or a chance finding.

The value‐based, age‐ and sex‐ adjusted HRQoL results of patients were normal, regardless of stratification by clinical manifestations of CAA (ICH, cognitive decline or TFNE). One explanation might be that our study included a relatively healthy sample because we excluded patients who were unable to fill out the questionnaires. Alternatively, patients might have adapted to, or learnt to accept their disabilities over time, causing their perceived HRQoL to normalize [34, 35]. This might be supported by fact that, across all groups in our study, ≥75% of patients had been diagnosed for ≥1 year and the median time since previous ICH was 3–4 years.

We found that 23% (95% CI 13%;33%) of patients with sCAA, 27% (95% CI 10%;44%) with mixed CAA‐HTA, and 14% (95% CI 5%;22%) with D‐CAA showed depressive symptomatology. Similarly, anxiety was present in 23% (95% CI 13%;33%) of patients with sCAA, 26% (95% CI 9%;43%) with mixed CAA‐HTA and 23% (95% CI 13%;34%) with D‐CAA. To put this in context, in the general Dutch population at the time of our study, the prevalence of mood disorders was 8.8%–10.8% and that of anxiety disorders was 13.8%–16.6%, both declining with age [36]. At the same time we note that, because our questionnaire‐based endpoint does not necessarily relate to a formal clinical psychiatric diagnosis from an extensive in‐person interview, these proportions should not be directly compared. When considering the previous literature, a previous sCAA study found depression (combination of the Geriatric Depression Scale, HADS and/or Depression Anxiety Stress Scale) in 35% of 77 patients (21% of non‐ICH patients; 46% of those with previous ICH) [37]. This aligns with the findings and CIs in our study. Similarly, our anxiety prevalence estimates align with that same previous study (36%, regardless of history with ICH). Additionally, our findings, are in line with estimations in informant‐reported studies, reporting depression in 15%–49% and anxiety in 7%–38% (all measured with the neuropsychiatric inventory questionnaire) of patients with sCAA [8, 9, 10]. Although none of these studies, including our study, confirmed psychiatric diagnoses, the testing accuracy of the CES‐D and HADS to screen for major depression and for anxiety disorders with the used cut‐off value is high, and expected to minimize the number of false positives [21, 24, 38]. Therefore, clinicians should be vigilant of these symptoms, as clinical depression and anxiety disorders can be treatable.

A strength of our study is that we gained knowledge of the disease burden and the impacted domains of life for patients in a large sample, with a high response rate, that largely covers the CAA disease spectrum. Moreover, by including patients with presymptomatic D‐CAA we were able to capture information about the early phase of CAA in a relatively ‘pure’ form of CAA, and by including mixed CAA‐HTA we increased applicability of our findings to regular clinical practice.

We also note some study limitations. First, no disease‐specific HRQoL questionnaire exists. This limits the recognition of CAA‐specific impairments which might not be captured by the EQ‐5D‐5L or SF‐36. Second, our study did not include a control group. We mitigated this limitation by contrasting our findings to widely used normative data, and to large, up‐to‐date reference data from a large national mental health survey study [36]. However, we note that the normative scores for HRQoL and mood might in general have increased or shifted over time since their publication due to (non‐) medical advances (Dutch EQ‐5D norms published in 2014; SF‐36 norms in 1998). This might have caused our study to have missed differences in HRQoL between patients and healthy persons, which might in future studies be circumvented by sampling healthy controls. Third, response bias might limit the generalizability of our findings. We have selected a relatively healthy sample because patients who were unable to fill out the questionnaires were excluded and because patients with an avoiding coping strategy might have declined participation. Finally, our center is a national referral center. As such, we often see a selected sample of more complex cases, as well as less severe cases of CAA, including patients who seek a single consultation for a more detailed explanation of their disease. This might have biased our estimates towards better HRQoL. The influence of sampling and differing disease severities might best be assessed in replication studies.

In conclusion, CAA has an impact on emotional role functioning, social functioning, vitality and usual activities, irrespective of CAA being sporadic, with concomitant hypertensive arteriopathy or the Dutch‐type hereditary variant, and irrespective of CAA‐related medical history (ICH, cognitive decline, or TFNE). In addition, mood disturbances affect one sixth to one quarter of patients with CAA. Our results underline the importance of continued research into disease‐modifying and supportive (psychosocial) treatments to alleviate the global burden of CAA. As rates of ICH recurrence or cognitive decline are challenging, if not infeasible, endpoints for clinical CAA trials, one might argue for the inclusion of improvement on specific domains of HRQoL as either primary or secondary endpoints in clinical trials. However, before this can be done, the role limitations imposed by CAA should be explored in qualitative studies.

## AUTHOR CONTRIBUTIONS

**Kanishk Kaushik:** Conceptualization; investigation; writing – original draft; methodology; visualization; formal analysis; project administration; data curation. **Natasha G. Waslam:** Writing – review and editing; formal analysis; data curation; investigation; writing – original draft; visualization. **Reinier G. J. van der Zwet:** Investigation; writing – review and editing; project administration; visualization. **Sabine Voigt:** Investigation; methodology; writing – review and editing. **Rosemarie van Dort:** Project administration. **Erik W. van Zwet:** Methodology; formal analysis. **Gisela M. Terwindt:** Methodology; writing – review and editing; supervision. **Ellis S. van Etten:** Supervision; methodology; writing – review and editing. **Marieke J. H. Wermer:** Writing – review and editing; resources; supervision; investigation; conceptualization; methodology; funding acquisition.

## FUNDING INFORMATION

Clinical Established Investigator grant of the Netherlands Heart Foundation 2016 T086 to Marieke J. H. Wermer. The funding agencies had no role in designing or conducting this study.

## CONFLICT OF INTEREST STATEMENT

Marieke J. H. Wermer reports independent support from the Dutch Heart Foundation (Clinical Established Investigator grant 2016T86) and the Dutch Brain Foundation. Gisela M. Terwindt reports independent support from the Dutch Research Council (NWO memorable BIONIC 733050822), the Dutch Heart Foundation, the Dutch Brain Foundation, and the Dutch CAA foundation. All other authors report no disclosures or competing interests.

## Supporting information


Appendix S1:

## Data Availability

The data that support the findings of this study are available from the corresponding author upon reasonable request. The data are not publicly available due to their containing information that could compromise the privacy of research participants.
