# Supplementary material for: Quality of life, depression and anxiety in cerebral amyloid angiopathy: A cross‐sectional study
Source: Eur J Neurol. 2024 Sep 22;31(12):e16476. doi: 10.1111/ene.16476 (PMC11555154; doi:10.1111/ene.16476)
Supplement: Supplementary file 1 — Appendix S1: [file ENE-31-e16476-s001.docx]

**SUPPLEMENTAL MATERIAL**

### **Supplemental Methods**

Clinical data collection

Time since diagnosis was recorded as the difference between questionnaire response and date of first presentation due to CAA-related complaints, or date of genetic APP-mutation confirmation. Time since previous symptomatic ICH was recorded as the difference between questionnaire response and date of last recorded clinical presentation of focal neurological deficits with imaging confirmed ICH (i.e. symptomatic ICH) in the patient file. Permissions for the academic use of the EQ-5D-5L was obtained from the EuroQOL Group (Registration:39323).

Choice of questionnaires

While disease-specific questionnaires better allow evaluation of outcomes related to the particular CAA patient population, no such questionnaires exist for CAA. Because the disease consequences of CAA were expected to be different from stroke or Alzheimer’s Disease (for which disease-specific questionnaires do exist), we opted for two common generic validated questionnaires to measure HRQoL.

Because administrating a full psychiatric interview was infeasible for this study, we opted for two screening questionnaires to assess depression. To best capture the spectrum of complaints that might be present in clinical depression, we administered both the CES-D and HADS, which give differing weights to somatic and non-somatic complaints.

Handling of missing data

In case of missingness of demographics, medical history or clinical symptoms at the most recent outpatient clinic visit, we assumed informative-missingness and collected dichotomous information about having a medical history of symptomatic ICH, hypertension, diabetes mellitus, malignancies, allergies, gastro-intestinal disease, pulmonary disease, eye disease from previous medical correspondence. Patients with presymptomatic D-CAA often present for genetic consultation and diagnosis, after which they return for regular medical care upon onset disease-related complaints. Therefore, we assumed informative-missingness with regards to neurological history for these patients.

Missing data on the SF-36 were handled according to the manual: if a respondent answered ≥50% of the questions for one scale, mean imputation of that scale was done for the missing items. We performed subject-mean imputation for the CES-D and HADS, assuming missingness at random in questionnaires with ≤20% missing items.^26,27^ Questionnaires with >20% missing responses were assumed to be missing not at random and omitted from the analysis. To assess the influence of this imputation, we performed a sensitivity analysis for the CES-D and HADS in which we only used complete cases.

Linear regression modelling assumptions were tested and not violated. Analyses were performed using R (version 4.3.1). Statistical significance was considered at α=<0.05.

### **Supplemental Results**

SF-36

After controlling for age and sex, patients with mixed CAA-HTA scored worse than the norm population on the domains of role functioning due to emotional problems (adj.β[95%CI]: -16[-31;-1]), vitality (adj.β[95%CI]: -10[-18;-2]) and social functioning (adj.β[95%CI]: -9[-17;-1]).

Sensitivity analyses

The baseline characteristics of in- and excluded patients were similar, although fewer excluded patients with sCAA had a history of ICH (30% vs 47%) and fewer excluded patients with D-CAA had cognitive decline (18% vs 35%). The proportions of patients recruited via post or e-mail did not differ between in- and excluded patients.

### **Table S1:** SF-36 health profile of patients with sporadic CAA, stratified by history of ICH, cognitive decline and TFNE.

|  | **ICH+**  **(n=34)** | **ICH-**  **(n=40)** | **Cognitive decline +**  **(n=29)** | **Cognitive decline -**  **(n=45)** | **TFNE+**  **(n=21)** | **TFNE-**  **(n=53)** |
| --- | --- | --- | --- | --- | --- | --- |
| SF-36 subscales, mean(SD) |  |  |  |  |  |  |
| Physical Functioning | 74 (20) | 79 (24) | 77 (23) | 76 (21) | 78 (23) | 76 (21) |
| Role Limitations due to Physical Problems | 48 (41) | 60 (40) | 58 (42) | 52 (40) | 54 (42) | 55 (41) |
| Role Limitations due to Emotional Problems | 70 (39) | 63 (43) | 58 (46) | 71 (37) | 63 (44) | 67 (40) |
| Vitality | 66 (21) | 60 (22) | 58 (21) | 65 (21) | 61 (19) | 63 (22) |
| Mental Health | 76 (18) | 70 (19) | 67 (17) | 76 (20) | 73 (15) | 73 (21) |
| Social Functioning | 73 (22) | 70 (22) | 67 (20) | 74 (22) | 74 (19) | 70 (23) |
| Bodily Pain | 80 (22) | 75 (25) | 74 (27) | 79 (21) | 74 (22) | 79 (24) |
| General Health | 62 (22) | 53 (25) | 49 (21) | 63 (24) | 53 (22) | 59 (25) |
| Health change | 45 (27) | 59 (23) | 55 (24) | 51 (27) | 55 (25) | 51 (27) |
| ICH intracerebral hemorrhage; TFNE transient focal neurological episodes | | | | | | |

### **Table S2:** SF-36 health profile of patients with Dutch-type CAA, stratified by history of ICH, cognitive decline and TFNE.

|  | **Symp-tomatic**  **(n=33)** | **Presymp-tomatic (n=29)** | **Cognitive decline +**  **(n=23)** | **Cognitive decline -**  **(n=39)** | **TFNE+**  **(n=4)** | **TFNE-**  **(n=58)** |
| --- | --- | --- | --- | --- | --- | --- |
| SF-36 subscales, mean(SD) |  |  |  |  |  |  |
| Physical Functioning | 69 (27) | 82 (15) | 76 (22) | 74 (24) | 68 (43) | 75 (22) |
| Role Limitations due to Physical Problems | 52 (46) | 75 (36) | 63 (46) | 61 (43) | 50 (58) | 63 (43) |
| Role Limitations due to Emotional Problems | 66 (44) | 67 (42) | 64 (44) | 68 (43) | 50 (43) | 67 (43) |
| Vitality | 60 (21) | 59 (16) | 58 (18) | 60 (20) | 68 (22) | 59 (19) |
| Mental Health | 75 (19) | 73 (14) | 70 (17) | 78 (16) | 80 (19) | 74 (17) |
| Social Functioning | 74 (22) | 80 (19) | 73 (23) | 79 (20) | 88 (10) | 75 (22) |
| Bodily Pain | 69 (23) | 74 (23) | 73 (22) | 69 (24) | 68 (37) | 71 (22) |
| General Health | 57 (21) | 65 (16) | 59 (18) | 61 (21) | 53 (22) | 60 (19) |
| Health change | 46 (28) | 46 (21) | 38 (22) | 50 (26) | 56 (13) | 45 (26) |
| ICH intracerebral hemorrhage; TFNE transient focal neurological episodes | | | | |  |  |

### **Table S3:** Comparison of HRQOL and depression summaries by diagnosis (sporadic or Dutch-type cerebral amyloid angiopathy) and medical history

|  | **ICH+** | **ICH-** | **Cognitive decline+** | **Cognitive decline-** | **TFNE+** | **TFNE-** |
| --- | --- | --- | --- | --- | --- | --- |
| **sCAA** |  |  |  |  |  |  |
| Responded EQ-5D/ Depression, N | **33/29** | **40/37** | **28/25** | **45/41** | **21/17** | **52/49** |
| EQ-VAS* | 79 (15) | 74 (18) | 73 (17) | 78 (16) | 78 (14) | 76 (18) |
| EQ-Index* | 0.85 (0.11) | 0.80 (0.18) | 0.79 (0.17) | 0.84 (0.14) | 0.84 (0.11) | 0.82 (0.17) |
| Depression† | 4 (14, 1-26) | 11 (37, 15-44) | 8 (32, 14-50) | 7 (17, 6-29) | 3 (18, 0-36) | 12 (25, 12-37) |
| **Mixed CAA-HTA** |  |  |  |  |  |  |
| Responded EQ-5D/ Depression, N | **10/10** | **19/16** | **14/13** | **15/12** | **2/2** | **27/23** |
| EQ-VAS* | 83 (10) | 72 (22) | 68 (25) | 83 (10) | 83 (4) | 76 (20) |
| EQ-Index* | 0.87 (0.11) | 0.80 (0.26) | 0.77 (0.30) | 0.87 (0.11) | 1 [NA] | 0.81 (0.22) |
| Depression † | 1 (10, 0-29) | 6 (38, 14-61) | 6 (46, 19-73) | 1 (8, 0-24) | 0 (0) | 7 (30, 12-49) |
| **D-CAA** |  |  |  |  |  |  |
| Responded EQ-5D/ Depression, N | **33/29** | **33/30** | **24/22** | **42/36** | **4/4** | **62/54** |
| EQ-VAS* | 77 (17) | 78 (12) | 74 (14) | 79 (15) | 79 (22) | 77 (15) |
| EQ-Index* | 0.81 (0.18) | 0.86 (0.14) | 0.81 (0.17) | 0.86 (0.16) | 0.73 (0.31) | 0.85 (0.15) |
| Depression † | 3 (10, 0-30) | 5 (17, 3-30) | 2 (9, 0-21) | 6 (17, 4-29) | 0 (0) | 8 (15, 5-24) |
| CAA cerebral amyloid angiopathy; D-CAA Dutch-type CAA; HTA hypertensive arteriopathy; ICH intracerebral hemorrhage; sCAA sporadic CAA; TFNE transient focal neurological episodes; VAS visual analogue scale  * mean(SD)  † n(%, 95%CI)  *Depression: Medical history of depression or use of anti-depressive medication, and high score on the CES-D and/or HADS-D; or high score on both CES-D and HADS-D.* | | | | | | |

### **Table S4:** Characteristics of excluded and non-responding patients

|  | **sCAA** | **Mixed CAA-HTA** | **D-CAA** |
| --- | --- | --- | --- |
|  | **All**  **(n=40)** | **All**  **(n=24)** | **All**  **(n=28)** |
| Age, years, mean(SD) | 74 (8) | 71 (6) | 57 (14) |
| Men, n(%) | 18 (45) | 11 (46) | 12 (43) |
| **Medical History, n(%)** |  |  |  |
| Symptomatic ICH | 12 (30) | 9 (38) | 17 (61) |
| Cognitive decline | 11 (28) | 15 (63) | 5 (18) |
| TFNE | 6 (15) | 4 (17) | 1 (4) |
| Epilepsy | 5 (13) | 4 (17) | 5 (18) |
| Hypertension | 18 (45) | 18 (75) | 10 (36) |
| Previous sICH count, median [IQR] | 0 [0-1] | 0 [0-1] | 1 [0-2] |
| **Elapsed time since event, median[IRQ]** |  |  |  |
| Diagnosis, years | 1 [0-3] | 1 [0-1] | 4 [2-9] |
| Last outpatient clinic visit, months | 7 [6-19] | 21 [16-22] | 29 [0-41] |
| Previous sICH*, years | 3 [3-4] | 2 [2-2] | 2 [2-4] |
| **Recruitment method, n(%)** |  |  |  |
| E-mail | 27 (73) | 9 (43) | 20 (71) |
| Post | 10 (26) | 12 (57) | 8 (29) |
| **Self-reported history of depression, n(%)** | 5 (13) | 1 (4) | 7 (25) |
| **Reason excluded, n(%)** |  |  |  |
| Non-responder | 14 (15) | 8 (9) | 7 (8) |
| Does not want to participate in research | 16 (17) | 7 (8) | 11 (12) |
| Participation too confronting | 4 (4) | 3 (3) | 3 (3) |
| Permanent disability | 6 (7) | 6 (7) | 7 (8) |
| CAA Cerebral amyloid angiopathy; D-CAA Dutch-type CAA; HTA hypertensive arteriopathy; sCAA sporadic CAA; (s)ICH (symptomatic) intracerebral hemorrhage; TFNE transient focal neurological episode(s)  * Recorded only for patients with history of symptomatic ICH. | | | |

### **Table S5:** Complete case analysis of HADS and CES-D

|  | **sCAA**  **(n=77)** | **Mixed CAA-HTA**  **(n=31)** | **D-CAA**  **(n=71)** |  |
| --- | --- | --- | --- | --- |
| CES-D, responded, n(% of all in group) | 58 (75) | 22 (71) | 58 (82) |  |
| Total score, median[IQR] | 11[4-8] | 15[9-20] | 8[5-18] |  |
| Scores high, n(%) | 21 (36) | 10 (45) | 16 (28) |  |
| HADS-D, responded, n(% of all in group) | 65 | 27 | 59 |  |
| Total score, median[IQR] | 4 [2-8] | 5 [2-8] | 4 [1-6] |  |
| Scores high, n(%) | 18 (28) | 7 (26) | 7 (12) |  |
| HADS-A, responded, n(% of all in group) | 66 | 26 | 58 |  |
| Total score, median[IQR] | 4 [2-7] | 5 [3-8] | 5 [2-7] |  |
| Scores high, n(%) | 16 (24) | 7 (27) | 14 (24) |  |
| CAA cerebral amyloid angiopathy; CES-D Center for Epidemiological Studies Depression scale; D-CAA Dutch-type CAA; HADS Hospital Anxiety and Depression Scale (-A for anxiety, -D for depression); HTA hypertensive arteriopathy; sCAA sporadic CAA | | | | |

### **Figure S1: Flowchart of study inclusions**


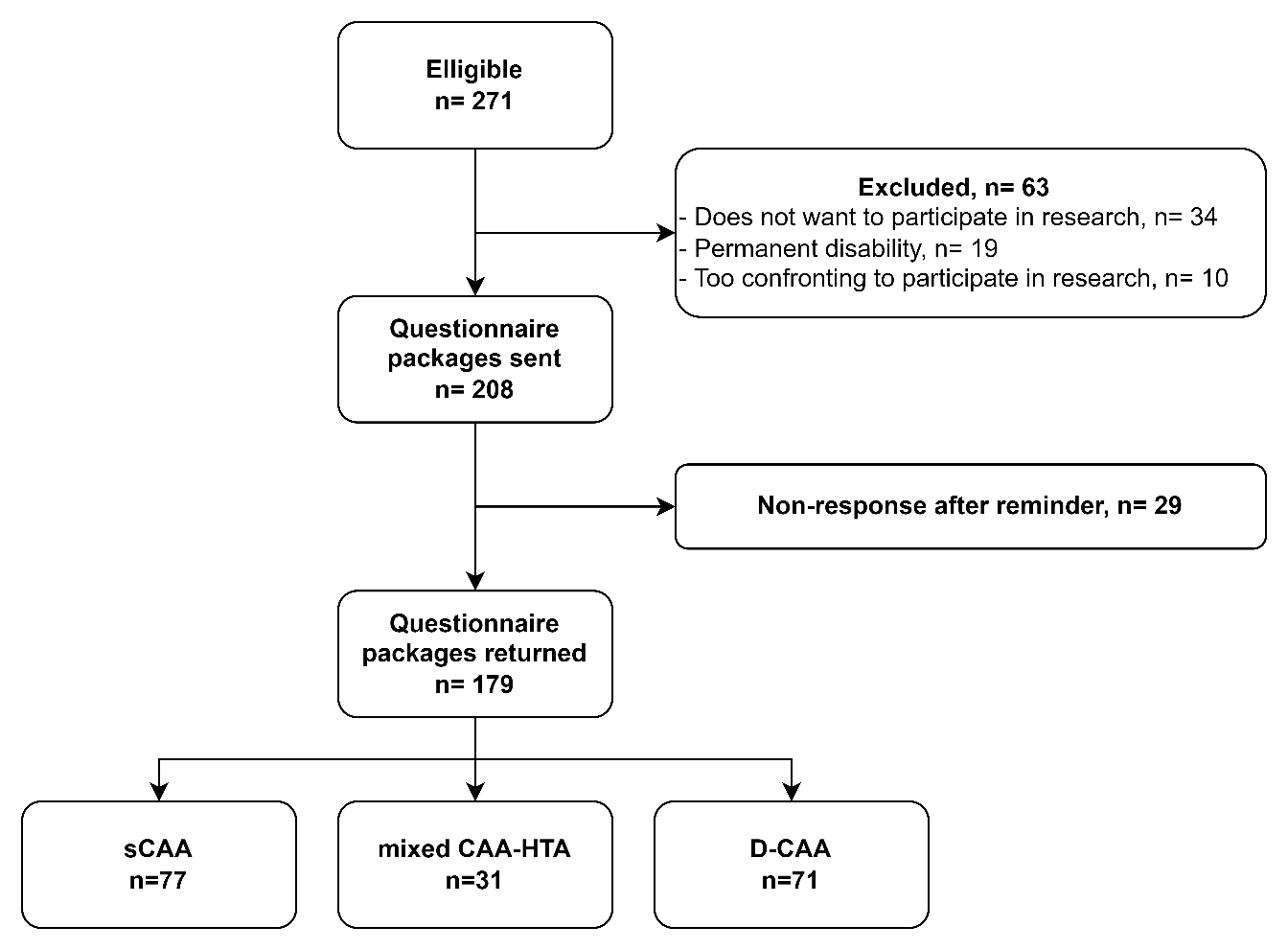


### **Figure S2:** Domain-comparisons of the SF-36 scores in patients with sCAA and Dutch-type (D-)CAA, stratified by history of intracerebral haemorrhage (ICH).


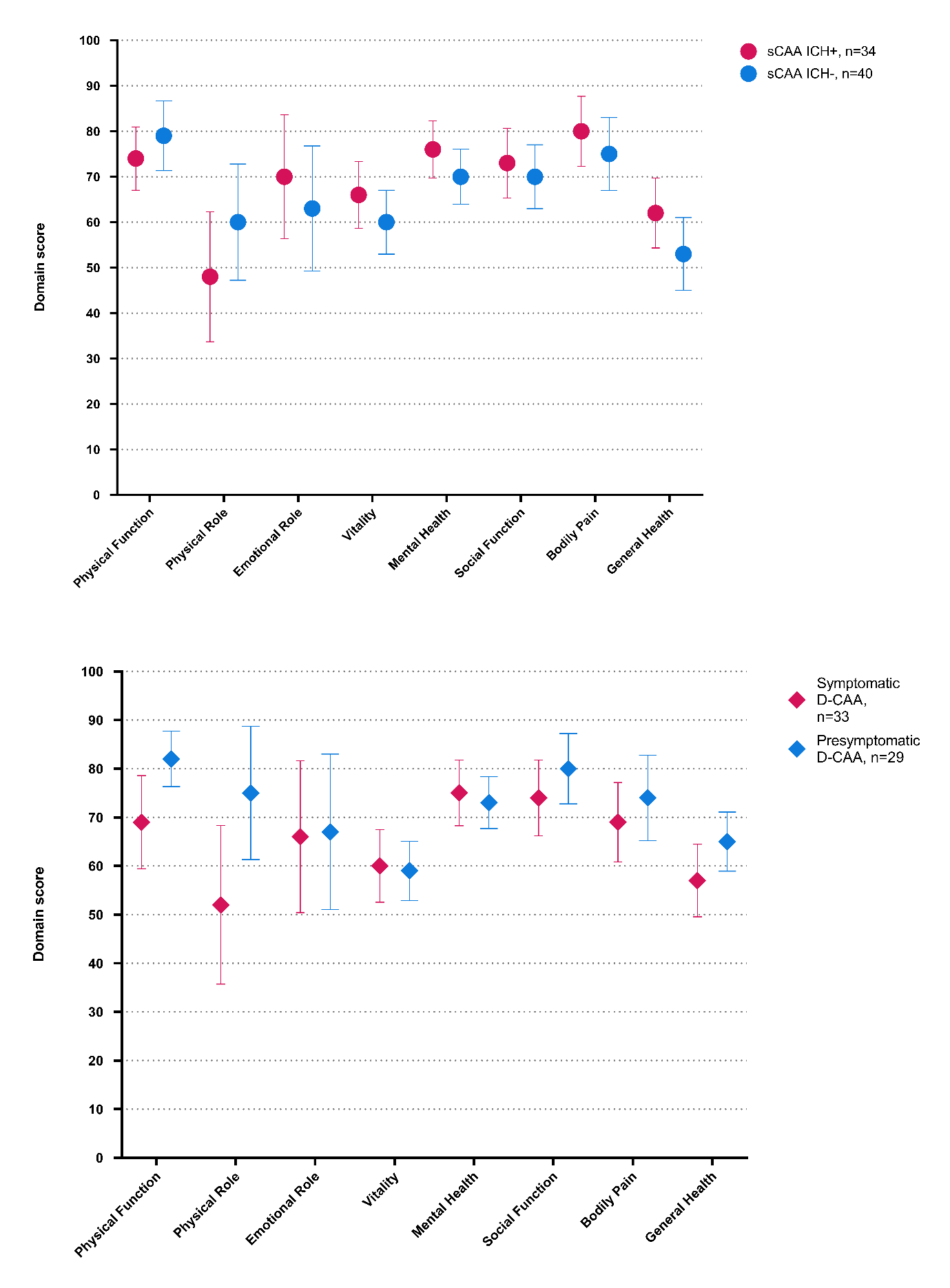


Plotted as mean(95%CI)

### **Figure S3:** Domain-comparisons of the SF-36 scores in patients with sCAA and Dutch-type (D-)CAA, stratified by history of cognitive decline.


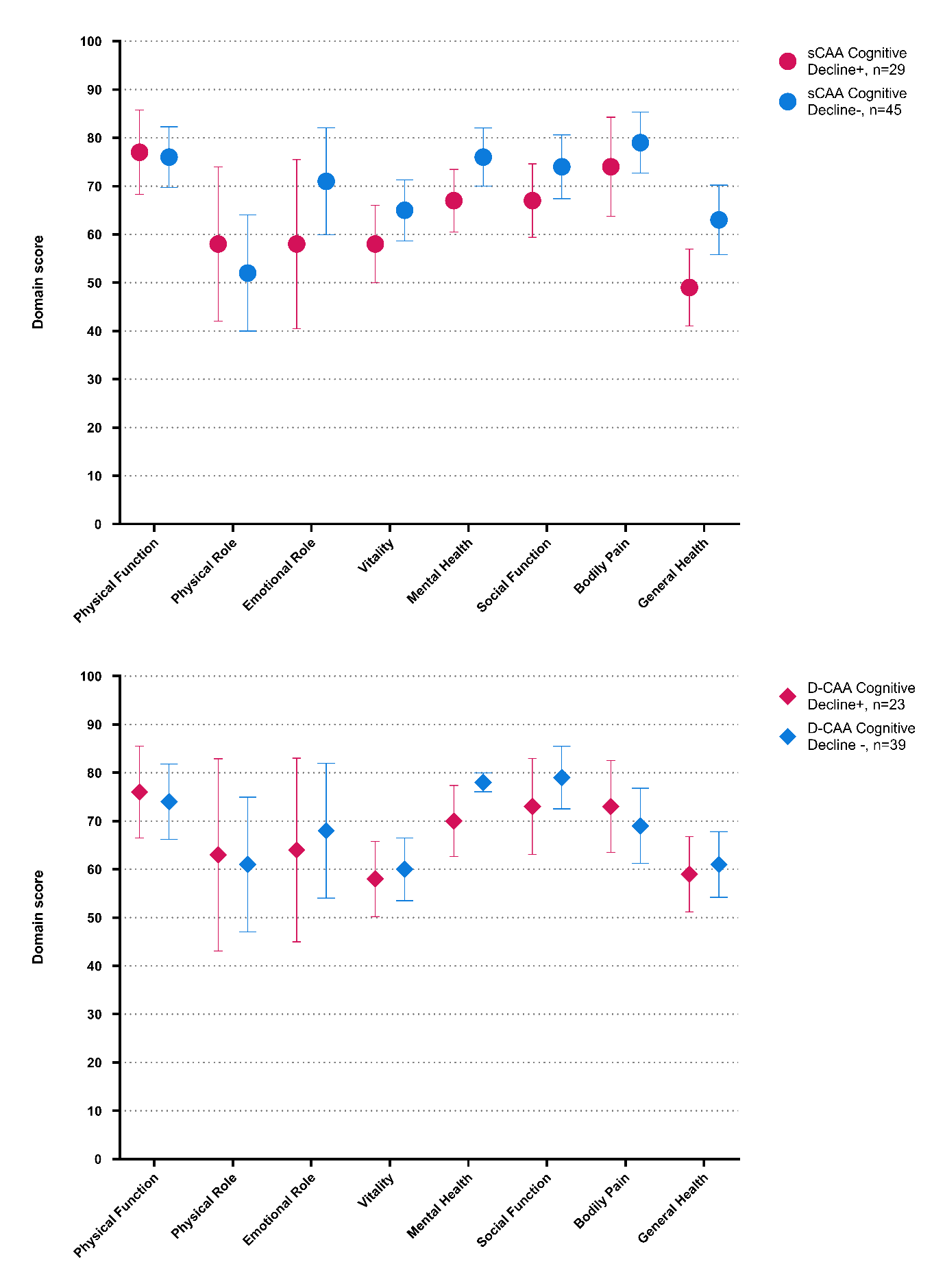


Plotted as mean(95%CI)

### **Figure S4:** Domain-comparisons of the SF-36 scores in patients with sCAA, stratified by history of transient focal neurological episodes (TFNE).


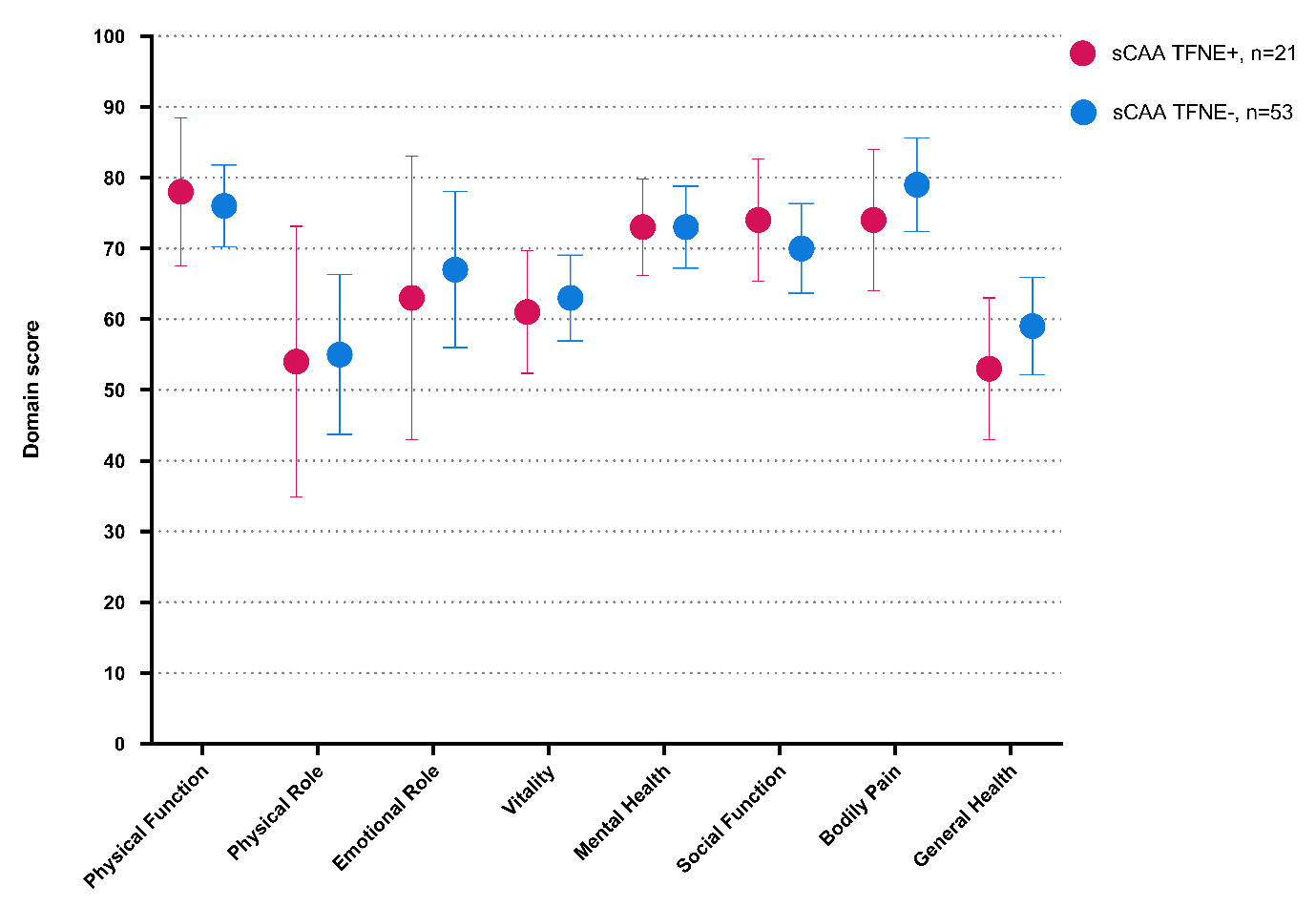


Plotted as mean(95%CI); D-CAA not shown due to limited number with history of TFNE (n=4).

### **Figure S5:** The EQ-5D-5L health profile of patients with sCAA and Dutch-type (D-)CAA, stratified by history of intracerebral haemorrhage (ICH)


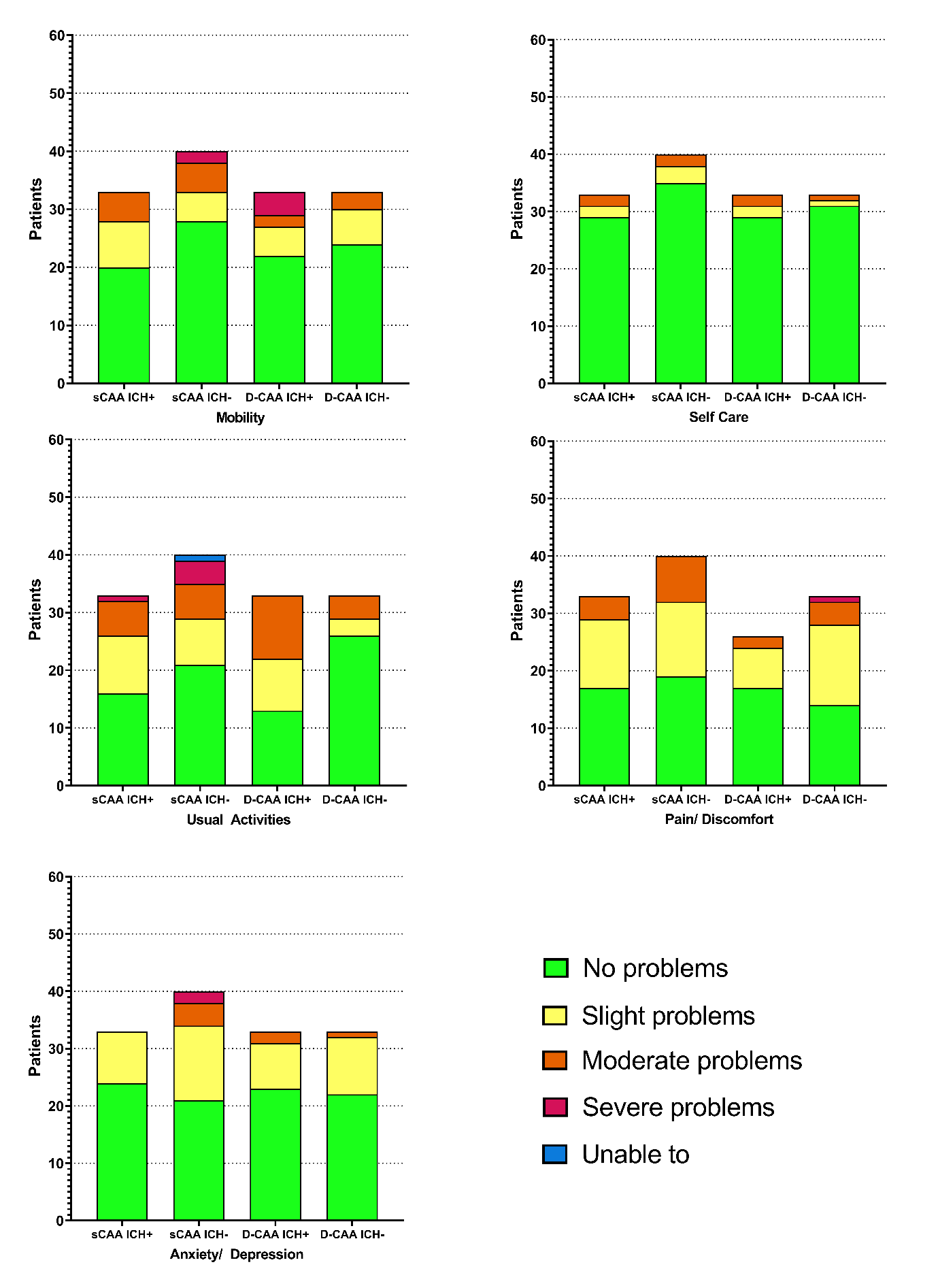


### **Figure S6:** The EQ-5D-5L health profile of patients with sCAA and Dutch-type (D-)CAA, stratified by history of cognitive decline (CD)


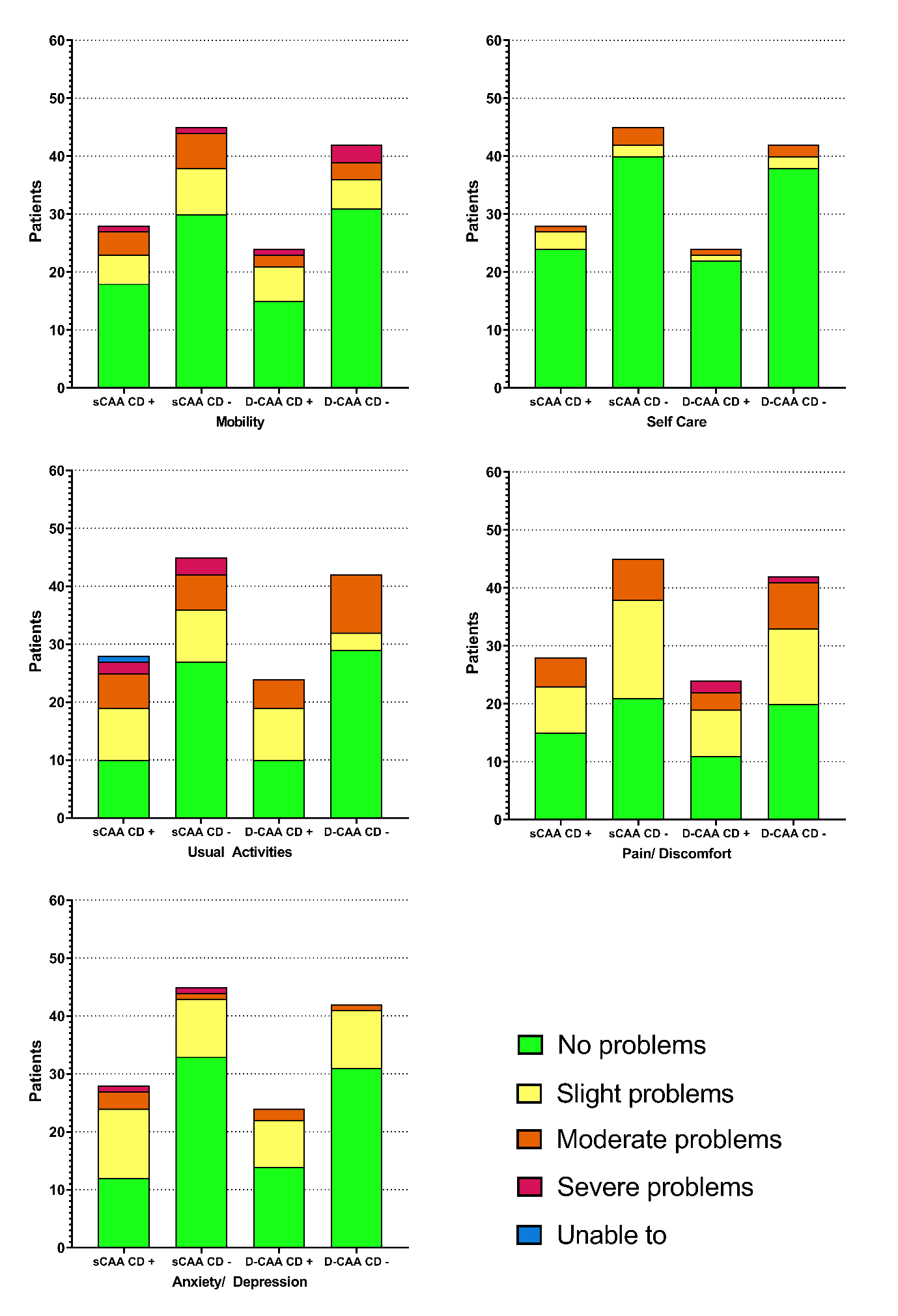


### **Figure S7:** The EQ-5D-5L health profile of patients with sCAA stratified by history of transient focal neurological episodes (TFNE).


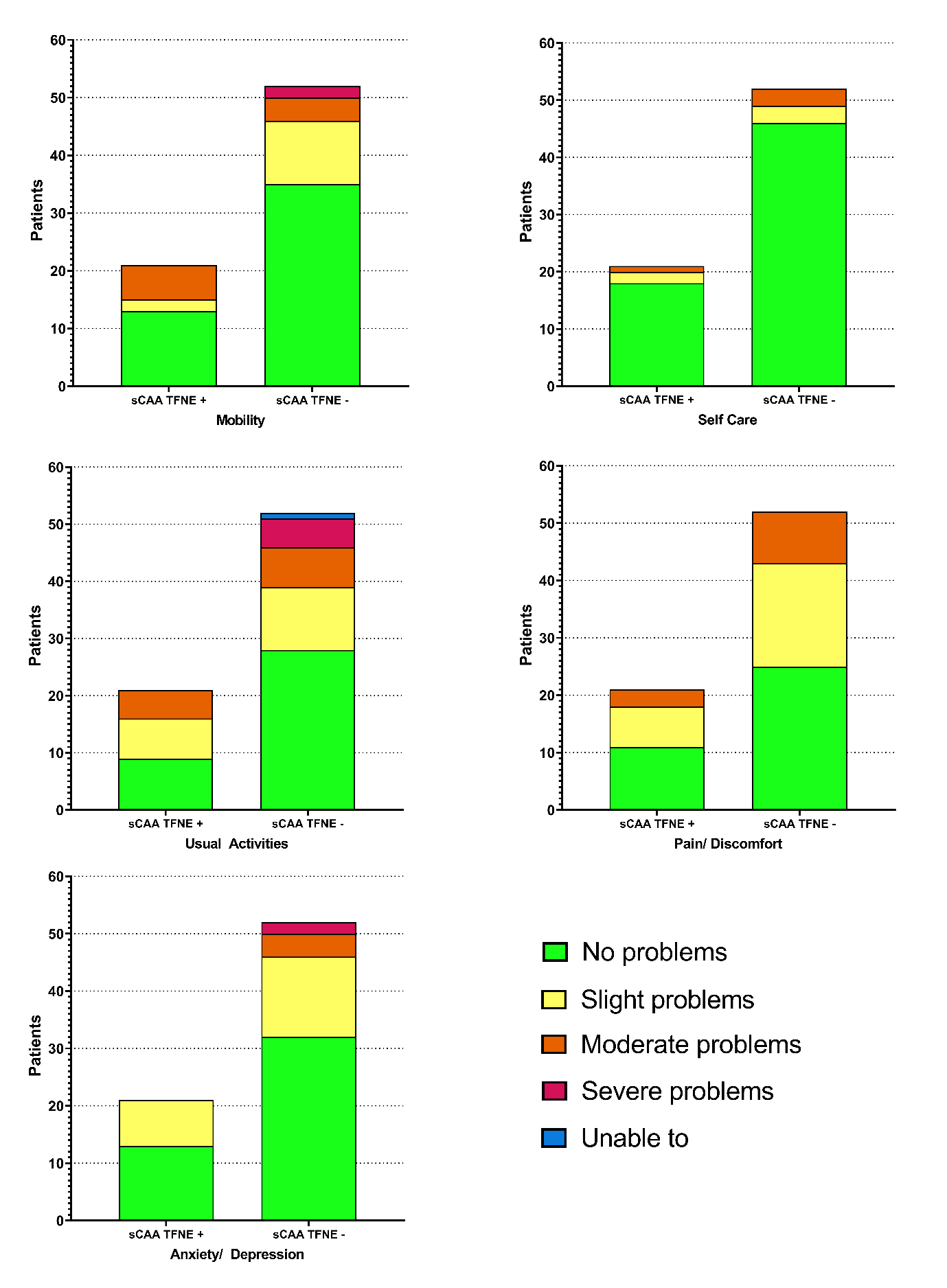


D-CAA not shown due to limited number with history of TFNE (n=4).
